# The Antimicrobial Effects of Coffee and By-Products and Their Potential Applications in Healthcare and Agricultural Sectors: A State-of-Art Review

**DOI:** 10.3390/microorganisms13020215

**Published:** 2025-01-21

**Authors:** Rosa Castro-Díaz, Norma Patricia Silva-Beltrán, Nohemi Gámez-Meza, Kadiya Calderón

**Affiliations:** 1 Departamento de Investigaciones Científicas y Tecnológicas, Universidad de Sonora, Hermosillo C.P. 83000, Sonora, Mexico; a215202244@unison.mx; 2 Department of Environmental Science, Water Energy Sustainable Technology (WEST) Center, University of Arizona, Tucson, AZ 85745, USA; normasilva@arizona.edu

**Keywords:** *Coffea arabica*, *Coffea canephora*, coffee by-products, antimicrobial activity, sustainable agriculture

## Abstract

Coffee is one of the most consumed beverages around the world. Its production is dominated by the species *Coffea arabica* and *Coffea canephora*. However, the coffee elaboration process leads to generating a significant amount of waste, which arises in various stages of coffee bean processing and is rich in natural bioactive compounds such as phenolic compounds and alkaloids. Particularly, chlorogenic and caffeic acids have a high antimicrobial potential and have been demonstrated to be effective against bacteria and viruses of healthcare and food relevance, including multi-resistant pathogens. However, the production and accumulation of coffee waste have a negative environmental impact since they can contaminate the surrounding environment due to the presence of organic molecules such as caffeine and tannins. In this context, exploiting natural resources as a source of compounds with the antimicrobial potential of, for example, the bioactive compounds obtained from coffee, has been evaluated in previous works. This review aims to summarize the current knowledge on the antimicrobial properties of coffee and its by-products and their potential application in the healthcare sector and disease control in agricultural crops, with particular emphasis on improving sustainability and efficiency in agriculture through making use of waste, which carries high importance in today’s society.

## 1. Introduction

Currently, coffee is one of the most consumed beverages, leading the global market with an estimated production, in the year 2021, of 168.6 million 60 kg bags, with a global consumption of up to 175.5 million bags. Coffee has also been used for medicinal purposes and added to a wide variety of cosmetic products. In the agricultural sector, coffee has been used as an organic fertilizer due to its nutrient content [1,2,3]. The main producers are Latin American countries such as Colombia and Brazil, Asian countries such as Indonesia and Vietnam, and African countries such as Ethiopia. However, Europe is considered the continent with the highest level of coffee beverage consumption, estimated at a third of the total global consumption of this product [2]. Coffee production is dominated by two species: *Coffea arabica*, which constitutes 58% of the world’s production, and *Coffea canephora*, colloquially known as Robusta coffee, which represents 42% of the estimated values for the years 2020–2021 [3].

The coffee elaboration process leads to the generation of a significant amount of waste, which arises in various stages of coffee bean processing. Initially, coffee beans represent 50% of the total weight of the fruit, while the remaining 50% consists of the husk, pulp, mucilage, and parchment. These by-products are the residues obtained during the primary processing of green beans. Once these beans undergo the roasting process, another waste known as silver skin is removed, which is a layer directly covering the coffee beans. Finally, during the preparation of coffee beverages, commercially in coffee shops or at home, a last residue called spent coffee grounds or coffee waste is produced [4].

The production and accumulation of spent coffee grounds have a negative environmental impact since they can contaminate the surrounding environment due to the presence of organic molecules such as caffeine and tannins. This is relevant since approximately 2 billion tons of this product is produced per year [2].

Within the broad spectrum of molecules found in spent coffee grounds, natural bioactive compounds such as phenolic compounds and alkaloids stand out. Additionally, during the coffee roasting process, certain molecules called melanoidins are formed, contributing to the coffee’s flavor. Collectively, these compounds confer to coffee antioxidant, neurostimulant, anti-inflammatory, and antimicrobial properties, among others. These properties are also present in this by-product obtained from coffee drink, since a significant amount of phytochemicals are retained within it [5].

One of the most important current challenges facing the healthcare sector is the development of antibiotic resistance by many bacteria, which limits the efficiency of the medications used daily for the treatment of a wide range of diseases. Antibiotic resistance also spreads in the agricultural sector, as the medications used to control diseases in crops are often the same as those used in humans [6]. This situation provokes the need to explore and exploit natural resources as a source of compounds with antimicrobial activity [7]. Research has shown that bioactive compounds extracted from coffee possess antimicrobial properties, demonstrating efficacy against various Gram-positive and Gram-negative pathogens of healthcare and nutritional relevance [8,9]. Additionally, the recent pandemic caused by the SARS-CoV-2 virus has increased the need to identify natural-origin compounds with antiviral activity. In this sense, studies have also been conducted on the potential of coffee against viruses such as influenza, herpes, and COVID-19, showing promising results [10,11,12].

This review examines the antimicrobial properties of coffee and their potential applications in the health sector and disease control in agricultural crops. It highlights the latest research findings, with a particular emphasis on the innovative use of spent coffee grounds. Key topics include the composition of coffee, its antimicrobial properties, and the potential of spent coffee grounds as a valuable resource.

## 2. Classification of Active Compounds in Coffee and Their Biological Effect

The coffee-producing plants belong to the Coffea genus of the Rubiaceae family. They are evergreen shrubs that typically reach an average height of 3 to 3.5 m, producing white flowers and the fruit known as cherry, which contains two seeds inside corresponding to the green coffee beans. This group comprises an estimated 103 species distributed across the continents of America, Africa, and Asia. Despite the high number of species, only two of them are currently cultivated for commercial purposes: *Coffea arabica* and *Coffea canephora* [13].

Despite coffee being consumed mainly for its flavor and neurostimulant activity, it presents properties that positively affect human health. These properties are conferred by the bioactive compounds, which come directly from the secondary metabolism of the plant of origin. Some of these molecules correspond to phenolic compounds, which originally played a protective role against diseases in the plant [14]. Phenolic compounds are characterized by the presence of a hydroxyl group attached to an aromatic ring, known as a phenol. These are further divided into simple phenolic compounds, which are characterized by having the simplest structure consisting of a single phenol group, and polyphenols, which are formed by more than one phenol unit [15].

### 2.1. Phenolic Acids

In coffee, the predominant phenolic compounds belong to the first group, mainly consisting of hydroxybenzoic acids and hydroxycinnamic acids. Among the hydroxybenzoic acids in coffee, compounds such as gallic acid and syringic acid are present (Figure S1) [16]. These types of compounds are characterized by the presence of a carboxyl functional group attached to the benzoic (aromatic) ring [15]. Chlorogenic, caffeic, ferulic, and p-coumaric acids are hydroxycinnamic acids that can be found in coffee [16,17]. This group is characterized by the presence of a carboxyl group, which is connected to the phenol group by a carbon–carbon double bond (C=C) [15]. The antimicrobial activity of coffee is predominately due to chlorogenic acids, which are the most relevant compounds. Chlorogenic acids are formed through the esterification of caffeic acid and quinic acid, with over 300 different types found in coffee [7,18].

Green coffee beans rank second in chlorogenic acid content, after yerba mate (*Ilex paraguariensis*). However, coffee is the main contributor to chlorogenic acid intake in the diet, with around 200 mg contained in 200 mL, equivalent to the content of an average cup of coffee, and being more abundant in *C. canephora*, reaching values of up to 8 g per 100 g of dry matter. The antimicrobial activities are attributed to chlorogenic acids [18,19].

In general, the total content of chlorogenic acids present in coffee is determined by the degree of ripeness of the green beans, the variety of coffee, and the geographic location of the cultivation origin [17]. Another important hydroxycinnamic acid due to its biological activity present in coffee is caffeic acid (Figure S2b). It is typically found as a simple ester with quinic acid or a saccharide. Like chlorogenic acids, caffeic acid has also been shown to have antimicrobial activity [20].

Within the group of polyphenols, coffee contains molecules such as flavonoids, specifically compounds like catechin, epicatechin, and quercetin (Figure S3) [21]. The antioxidant activity of coffee, characterized by its ability to neutralize free radicals in the body, is primarily attributed to these compounds. To a lesser extent, they also contribute to the antimicrobial activity of coffee and its derivatives [22,23]. Notably, catechin obtained from green tea has demonstrated a growth-inhibiting effect against pathogens such as *Streptococcus mutans* [24]. The antibacterial activity of quercetin obtained from various plant products against both Gram-negative and Gram-positive bacteria has also been reported, including an inhibitory effect on biofilm formation in pathogenic microorganisms [25].

### 2.2. Alkaloids

Another group of bioactive compounds represented in coffee are alkaloids, which are nitrogenous organic compounds with a heterocyclic structure [26]. The most well-known alkaloid in coffee is caffeine (Figure S4a), which is popular for being a central nervous system stimulant, making it one of its main attractions for the average consumer [1]. Caffeine is a purine-type alkaloid and is one of the compounds that gives bitterness to coffee. Like with chlorogenic acids, caffeine content in *C. canephora* beans is higher than in *C. arabica*, with approximately twice the concentration. However, the amount of caffeine present in coffee brews depends on factors such as the cultivation origin, the type of bean, and the method of beverage preparation [17]. Trigonelline (Figure S4b) is the second most abundant alkaloid in green coffee beans. It is a derivative of pyridine and is formed through enzymatic methylation of nicotinic acid. Like caffeine, it contributes to the flavor and aroma of coffee. Trigonelline has been found in greater quantities in Arabica coffee than in Robusta coffee; however, its concentration depends on factors such as coffee variety and environmental conditions in the cultivation region [1,17]. Various studies have attributed part of the antimicrobial activity found in coffee to these compounds. In the case of caffeine, it has been observed that when acting alongside a clinically used antibiotic, it can enhance its bactericidal activity against certain microorganisms [7,27,28].

During the roasting process of green coffee beans, many of the natural components of the plant are transformed, giving rise to new ones. Melanoidins are one of the compounds formed during this process, which are nitrogenous molecules of high molecular weight with a brown color, formed by a particular mechanism known as the Maillard reaction [29]. This reaction occurs during the processing of certain products at high temperatures and involves the reaction between a free amino group and the carbonyl group of a reducing carbohydrate [30]. Melanoidins are primarily composed of carbohydrates such as arabinogalactans and galactomannans, phenolic compounds, and proteins [29,31]. It has been demonstrated that coffee melanoidins exert significant antimicrobial effects against clinically and nutritionally relevant pathogens [32].

### 2.3. Terpens

In coffee, other bioactive compounds such as terpenes can also be found. Terpenes are characterized as monomers derived from another compound known as isoprene and are natural secondary metabolites of a wide variety of plants. They can be found in microorganisms, such as bacteria, fungi, and algae. These molecules are classified according to the number of isoprene rings present in their structure, resulting in compounds such as monoterpenes, diterpenes, and triterpenes [33].

Among the terpenes that can be found in coffee, diterpenes stand out. They are defined as pentacyclic alcohols formed by the union of four isoprene units on a 20-carbon skeleton. The most abundant diterpenes in coffee are kahweol and cafestol (Figure S5), with *C. arabica* presenting the highest content of both compounds [17]. An unfiltered cup of coffee usually contains 3 to 6 mg of these compounds [34]. Diterpenes are particularly attributed with antioxidant activity, as they exert a protective effect against damage caused by oxidative stress promoted by factors such as DNA itself and hydrogen peroxide. It has also been found that these molecules have an anti-inflammatory effect [17]. In plants, diterpenes have shown antifungal effects against phytopathogens such as *Colletotrichum gloeosporioides* and *Fusarium oxysporum*, with cafestol being particularly effective. This is one of their main activities as natural secondary metabolites, playing a protective role against fungal pests [35]. 

## 3. Chemical Composition of Coffee Extracts and By-Products

The bioactive compounds present in coffee have garnered the interest of many researchers primarily due to their potential biological activity and possible application in sectors such as the pharmaceutical, food, and agricultural industries, etc. To study these compounds, coffee or products derived from its production need to undergo an extraction process that allows for the recovery of the highest concentration of these substances [4,7]. The amount of bioactive compounds obtained from extraction processes depends on a wide variety of factors, ranging from the intrinsic characteristics of the plant of origin, such as species, variety, genetic composition, geographical location, and environmental and climatic conditions; cultivation methods; processing methods of the coffee beans, whether dry, wet, or semi-wet, to the type of product being worked with, whether it is a sample taken directly from the plant, such as leaves, or working with green or roasted coffee beans, or with by-products of their processing, such as pulp, mucilage, and bagasse, among others [17,36,37].

For example, the roasting of coffee beans leads to the generation of new compounds that were not naturally present in the plant but are the result of the interaction of its metabolites, which are physically and chemically modified, giving rise to others, such as melanoidins. However, some natural bioactive molecules, such as certain phenolic compounds, may also be lost or modified [38,39]. Additionally, the way coffee beverages are prepared often modifies the content of bioactive compounds present in them [17]. Moreover, experimental work factors also interfere, such as the solvent used for extraction, like water, ethanol, methanol, etc., and the extraction method, whether by agitation, heating, ultrasound, or others. All these factors determine the total content of the compounds, as well as their concentration, that can be obtained from coffee and its derivatives [4].

**Figure 1:**
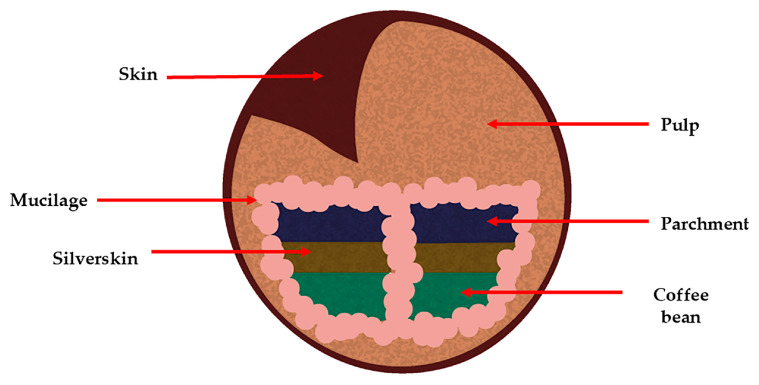
Internal composition of coffee cherry and by-products.

The enormous amount of waste produced by the coffee industry has led researchers to study it not only for its well-known biological activity but also to explore possible uses for the by-products generated during its processing. These by-products (Figure 1) evidently retain a considerable number of bioactive compounds that can be utilized, thereby also aiming to mitigate the negative environmental impact caused by their accumulation and improper handling [4].

Coffee By-Products

One of the first waste products that arises after processing green coffee beans is the pulp (Figure 1). This constitutes approximately 40 to 50% of the fruit’s mass, and its management after being removed from the cherry usually involves natural decomposition or use as fertilizer in the same coffee fields. However, this management often produces large amounts of wastewater and extremely unpleasant odors, thus constituting a negative impact on the surrounding environment [40,41]. This residue is composed of carbohydrates, soluble fibers, proteins, and minerals [4]. Coffee pulp has been shown to have a high content of bioactive compounds, mainly the following: chlorogenic acid, caffeine, epicatechin, and catechin [42]. Of the aforementioned compounds, chlorogenic acids constitute approximately 42% of the phenolic acids present in coffee pulp [7]. Other polyphenols, such as flavonols, flavan-3-ols, and pigments such as anthocyanidins, have also been reported (Table 1) [43]. The aqueous extract of coffee pulp has been reported to be effective against Gram-positive bacteria such as *Staphylococcus aureus* [40].

Mucilage (Figure 1) is another of the initial residues obtained when coffee beans are removed from the cherry, constituting around 14% of the fruit’s dry weight. It is removed from the beans through a fermentation process that usually lasts from 24 to 72 h. It is mainly composed of water, proteins, carbohydrates, and pectins and has a considerable content of phenolic compounds (Table 1) [44]. Mucilage is a waste generated in large quantities from coffee processing, with an estimated 56 kg of mucilage produced for every 60 kg bag of green coffee beans [45]. Among the bioactive compounds found in this product, chlorogenic acid and caffeine are noteworthy, particularly in ethanol extract. This same extract has been shown to be effective against Gram-positive bacteria such as *Bacillus cereus* [7].

The parchment (Figure 1) consists of the fibrous endocarp that covers the coffee beans and keeps them separated. It represents approximately 6.1% of the fruit, being found in lesser quantities than the pulp and mucilage [46]. This by-product is mainly composed of (α) cellulose, hemicellulose, lignin, and ash; however, it also has a significant content of bioactive compounds that remain even after being separated from the coffee cherry [47]. For every 100 kg of green coffee beans produced, approximately 18 kg of this residue is obtained [4]. Usually, this product is treated by producing pressed pellets used for energy generation, which are obtained or released by burning them. However, the improper management of this residue still represents a significant environmental threat, as it is necessary to find other ways to utilize or eliminate it more effectively and in line with the amount produced. This applies to any waste obtained from the coffee industry [46]. For parchment, phenolic compounds such as gallic acid, chlorogenic acid, p-coumaric acid, and sinapic acid, as well as alkaloids like caffeine, have been reported in an optimized (70%) ethanol extract (Table 1). These compounds have already been shown to contribute to the antimicrobial activity of coffee [47].

The combination of the three previously described residues, pulp, mucilage, and parchment, along with the skin, is known as coffee husk. This is the main waste obtained from the dry processing of green beans, representing approximately 50% of the fruit’s dry weight [4]. The coffee husk is mainly composed of carbohydrates, around 85%, and fibers and proteins. However, it has also been shown to have a significant number of bioactive compounds such as gallic acid, tannic acid, chlorogenic acid, epicatechin, and caffeine (Table 1) [48,49,50]. Usually, coffee husk residues are burned or used as compost; however, most of these are simply discarded without any specific or adequate treatment, which eventually becomes a source of environmental pollution [48,51].

On the other hand, silverskin (Figure 1) is a residue obtained during the roasting process of green coffee beans. It is a thin layer that directly covers the seeds [52]. This component of coffee is mainly composed of dietary fiber, polysaccharides, proteins, fats, and ash. However, it also has a significant number of bioactive compounds, mainly of an antioxidant nature, and represents approximately 4.2% of the coffee cherry [4,53]. In silverskin, bioactive compounds such as caffeine, trigonelline, 3-feruloylquinic acid, 5-caffeoylquinic acid, and 3-caffeoylquinic acid, which are isomers of chlorogenic acid, and p-coumaric acid have been observed in aqueous extracts obtained through a hydrodistillation process. Melanoidins can also be found since these are formed during the coffee roasting process, which is when this coating is removed, thus retaining some of these molecules (Table 1) [54,55].

Studies on coffee and its biological potential are not solely focused on extracts from derived products; they also analyze the properties and content of secondary metabolites present in both green and roasted coffee beans. The coffee roasting process alters the content and quantity of the compounds present. Therefore, understanding these differences helps to better understand which specific molecules can be obtained from both resources and what their most suitable application is. Additionally, the degree of roasting of the beans produces variations in the content of bioactive compounds [39]. Among many factors, the phytochemical composition of green coffee beans depends on their genetic load and degree of ripeness. It has been observed that the content of caffeine, trigonelline, and chlorogenic acids tends to be higher in green coffee [37]. In the case of chlorogenic acid, its concentration decreases during the roasting process due to exposure to high temperatures, resulting in the breakdown of the molecule into its components, caffeic acid and quinic acid. However, the degree of roasting also influences the content of chlorogenic acid. Light roasting retains a large amount of this compound, while darker roasting results in a lower concentration of chlorogenic acid [5]. Nevertheless, its presence after roasting remains significant, as it has been estimated that coffee provides the majority of chlorogenic acids in the diet in many regions, which is linked to the high consumption of this product [18]. Chlorogenic acids are estimated to represent 6 to 12% of the dry weight of green coffee seeds, making them the main source of chlorogenic acids in nature [18,56]. Alamri et al. (2022) found no significant differences in caffeine levels depending on the degree of roasting (light, medium, and dark), with values being quite similar among samples of Arabica coffee beans. Roasting beans leads to the formation of other bioactive compounds, such as melanoidins, which have been shown to have a wide range of biological properties [39]. Freitas et al. (2024) found a higher content of melanoidins in robusta coffee compared to *C. arabica*. Activity against Gram-positive and Gram-negative bacteria has been reported for both green and roasted coffee beans [38].

**Figure 2:**
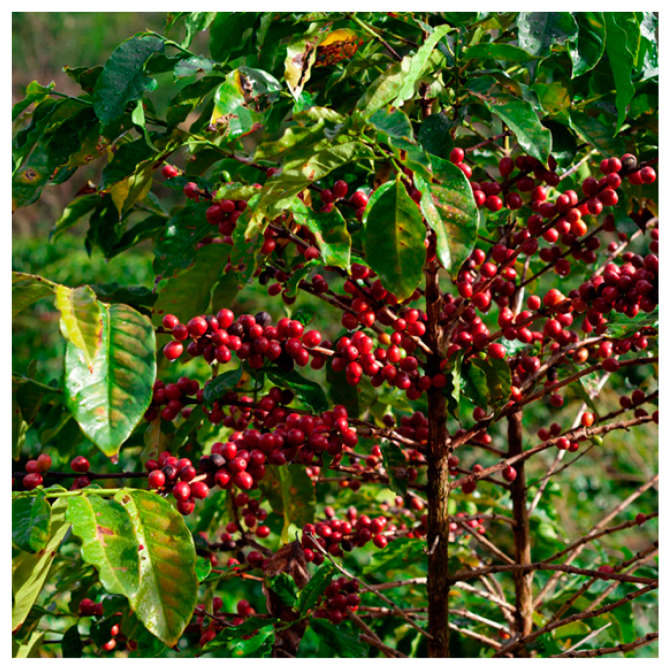
Coffee plant with mature cherries.

Extracts can also be obtained directly from the plant (Figure 2), such as from leaves. Yosboonruang et al. (2022) determined the presence of bioactive compounds such as chlorogenic acid and caffeine in a leaf extract of *C. canephora*, and they also analyzed its antibacterial properties against Gram-positive and Gram-negative bacteria. They were found to be particularly effective against *Bacillus subtilis* and *Staphylococcus aureus*, known pathogens transmitted through food [8].

Finally, the last residue produced in the coffee industry is the spent coffee grounds, as they are obtained directly from the preparation of coffee beverages. It is estimated that around 6 million tons of spent grounds are produced annually, with 50% coming from the commercial preparation of coffee in cafeterias and the remaining 50% from the industrial production of soluble coffee. This residue constitutes 90% of the initial coffee beans [2,52,57]. Spent grounds are mainly composed of polysaccharides, proteins, minerals, fats, dietary fiber, vitamin E, and lignin [16]. It has been reported that spent ground coffee extracts contain phenolic compounds such as chlorogenic, caffeic, gallic, ferulic, ellagic, p-coumaric, protocatechuic, and tannic acids, as well as catechin, epicatechin, quercetin, and rutin, and alkaloids such as trigonelline and caffeine when polar or moderately polar solvents, such as ethanol and methanol, are used in combination with deionized water (Table 1). It is also rich in melanoidins and diterpenes such as cafestol and kahweol [16,54,58,59,60]. Like many of the aforementioned residues, spent coffee grounds also retain significant amounts of environmentally harmful compounds, which can cause significant damage to soil and water near the deposition site, as organic compounds require large amounts of oxygen for decomposition. Badr et al. (2022) found that isopropanolic extract from spent grounds could inhibit the development of pathogenic bacteria such as *Escherichia coli* and fungi such as *Aspergillus*. Therefore, finding ways to reuse this product is a paramount task [2,61,62].

## 4. Antimicrobial Activities of Coffee and Its By-Products

Antimicrobials are compounds that have the ability to kill or inhibit the growth of microorganisms, such as fungi and bacteria [4]. As mentioned throughout this work, coffee has demonstrated a significant antimicrobial effect, and it is considered that this property is conferred by the bioactive compounds that compose it, mainly by the phenolic compounds and alkaloids described earlier. The following will present the antimicrobial mechanisms found in these compounds and will also mention the most recent research on the antimicrobial effect of coffee, as well as its derivatives, against bacteria, fungi, and viruses.

### 4.1. Antibacterial Activity of Coffee and Its By-Products

Canci et al. (2022) analyzed the antibacterial potential of an aqueous extract of roasted and green coffee (*C. arabica* and *C. canephora*) against pathogenic bacteria, calculating the minimum inhibitory concentration (MIC) of their extract and conducting a time kinetics test for microorganisms susceptible to the first test. In this case, they found that the bacteria sensitive to their extracts were *Salmonella typhimurium* and *E. coli*, both Gram-negative bacteria. However, no effect was observed on the bacteria *Lactobacillus plantarum* and *Lactobacillus rhamnosus*, which are known to be probiotic microorganisms. This may be due to the selective effect of the bioactive compounds, which can promote the growth of certain bacteria and inhibit the growth of others. This effect has been reported for chlorogenic acid and caffeic acid, both of which have been shown to have antibacterial and probiotic effects. In the case of the latter, it has been observed that the unabsorbed portion of these compounds serves as a substrate for beneficial bacteria inhabiting the gastrointestinal tract [63].

Yosboonruang et al. (2022) analyzed the antibacterial effect present in an aqueous extract of *C. canephora* leaf by calculating the MIC and conducting the agar well diffusion test. In this case, they found that their extract was particularly effective against *S. aureus* with an MIC of 6.25 mg/mL and for *Bacillus subtilis*, *E. coli*, and *S. typhimurium* with an MIC of 12.5 mg/mL for each of these strains. Other bacteria, such as *Bacillus cereus* and *Pseudomonas aeruginosa,* also proved susceptible, but at much higher concentrations, 50 and 25 mg/mL, respectively. These bacteria are known to be foodborne pathogens, so the effectiveness of coffee compounds as antibacterials may be useful in food preservative production. They also conducted a test to evaluate the integrity of the bacterial cell membrane, where they reported observing leakage of intracellular material, namely nucleic acids and proteins. The loss of these molecules inhibits protein production and DNA synthesis, eventually leading to the death of the microorganism [8].

Chaves-Ulate et al. (2023) tested the antimicrobial potential of an ethanolic extract of coffee mucilage on bacteria associated with the deterioration of certain foods and with human intestinal microbiota. In this case, the authors reported that their extract successfully inhibited the growth of Gram-positive bacteria such as *B. cereus*, *Listeria monocytogenes*, *Micrococcus luteus*, and *S. aureus*. However, the same result was not observed in Gram-negative bacteria such as *E. coli*, *Salmonella* sp., *Alcaligenes* sp., and *Pseudomonas* sp. The technique used to evaluate the antibacterial activity of the extract was agar microdilution, and the bacterium most susceptible to this extract was *B. cereus*. The authors discuss that the difference in the inhibition effect of the extract regarding bacterial groups may be due to the structural differences between the two types, as Gram-negative bacteria have an outer membrane with lipopolysaccharides, which provides greater protection against antibacterial compounds. It has been observed that some low molecular weight phenolic compounds have the ability to penetrate the cell membrane, causing the acidification of the cytoplasm, which consequently leads to bacterial death, making Gram-negative microorganisms more resistant to this mechanism. It is also considered that the inhibitory effect is closely related to the concentration of the extract, so at higher concentrations, not considered in this study, a different result could be observed [7].

Zubair (2024) analyzed the antibacterial activity of various aqueous extracts of coffee from C. arabica against biofilm-forming bacteria associated with diabetic foot ulcers. MIC tests showed that the most effective extract against strains of *P. aeruginosa*, *E. coli*, and *S. aureus* was powdered green coffee extract, with MIC values of up to 300 µL/mL. Tests were also conducted to observe the inhibitory effect on biofilm formation by coffee extracts using MIC sublevels (1/2xMIC and 1/16xMIC) in monocultures. Biofilm formation inhibition was evaluated using the quantitative method on flat-bottomed 96-well plates (microtiter plates). The powdered green coffee extract showed a biofilm inhibition level of 76% for *P. aeruginosa*, 62% for *E. coli*, and 53% for *S. aureus* on average. Meanwhile, roasted coffee bean extracts and spent coffee powder extracts yielded inhibition percentages of 66%, 53%, and 51%, respectively, for the same bacteria. For mixed cultures, the most effective sample was found to be the green coffee extract, with inhibition percentages of 66% (1/2xMIC), 56% (1/4xMIC), and 17% (1/8xMIC). Based on the previous results, it was determined that the ground green coffee extract was the most promising in terms of antibacterial activity, and therefore, it was selected for further tests. One of these tests consisted of evaluating membrane integrity; for this, an N-Phenyl-1-naphthylamine (NPN) uptake assay was conducted to evaluate the disruption of the bacteria’s outer membrane. Under normal conditions, the NPN compound cannot enter the cell due to the outer membrane; however, when this is disrupted, NPN can infiltrate the lipid bilayer, being detected through fluorescence emission, which increases when it is inserted into the membrane. In this case, the study reports an increase in NPN uptake of 51% in *P. aeruginosa*, 41% in *E. coli*, and 48% in *S. aureus*. In mixed strains, an average increase of 45% was observed. Disrupting the continuity of the outer membrane provides coffee with a highly important tool for inhibiting biofilm formation, as altering the integrity of the bacterial outer membrane may reduce its adhesion power and ability to bind to certain surfaces [64].

Jamalifar et al. (2019) studied the regulatory effects of green coffee extract on genes associated with the virulence of *P. aeruginosa*. This bacterium is notable for having strains resistant to various antibiotics, which they achieve through the action of two genes, las I and las R, which provide the microorganism with increased pathogenicity and more aggressive mechanisms against the affected person. This bacterium usually infects individuals with weakened immune systems, such as those suffering from conditions like cancer or AIDS. To collectively regulate the expression of these genes, these bacteria use the quorum sensing (QS) mechanism, which can lead to biofilm formation, toxin production, and other drug or antibacterial resistance systems. Therefore, one way to combat its development is to alter or stop this mechanism. In this study, the aim was to determine whether green coffee extract served as a good preventive measure for the expression of these genes, thus reducing the development and spread of antibiotic-resistant microorganisms. To determine this, a real-time PCR procedure was carried out, resulting in a 0.49 to 0.75 times reduction in the expression of these genes in the presence of green coffee extract, compared to the control samples, which did not receive the extract. This confirms the suspicion that the compounds present in coffee, particularly green coffee in this specific case, are capable of inhibiting biofilm development and other resistance mechanisms by decreasing the expression of the aforementioned genes. The authors attribute this antibacterial effect to phenolic compounds, particularly chlorogenic acid [65].

In a field similar to the previously analyzed study, Rawangkan et al. (2023) tested various aqueous extracts of coffee (*C. arabica*) to assess their antibacterial effect against multidrug-resistant strains of *E. coli* and also evaluated their potential as an adjuvant to the antibiotic ampicillin for treating diseases caused by this microorganism. Currently, ampicillin is not recommended for infections caused by *E. coli*, as most strains of this agent show resistance; therefore, this work focuses on finding a natural alternative that works on its own or can at least enhance the effect of the antibiotic. In this case, three different extracts were tested: dried green coffee beans, coffee pulp, and crude extracts from *C. arabica* leaves. These were subjected to MIC and minimum bactericidal concentration (MBC) tests individually on 28 multidrug-resistant *E. coli* strains. It was found that the most effective extract against these microorganisms was coffee pulp, with MICs ranging from 12.5 mg/mL to 50 mg/mL. Subsequently, a test was conducted to evaluate the combined effect of each coffee extract with ampicillin. In these analyses, it was found that only the combination of coffee pulp extract and ampicillin was effective at the antibacterial level, yielding an MIC of 0.02 mg/mL, while the MIC of the drug alone was 50 mg/mL, demonstrating that an agro-industrial byproduct of coffee production, such as pulp, in combination with an antibiotic, is a promising alternative to combat diseases caused by multidrug-resistant bacteria. To reinforce the previous findings, a bactericidal kinetics test was conducted, comparing the pulp extract and the antibiotic separately, as well as in combination. It was found that coffee pulp extract exerts bactericidal activity at concentrations of 50 mg/mL and 100 mg/mL in 20 and 7 h, respectively. Ampicillin, on the other hand, had a bactericidal effect after 4 h at a concentration of 50 mg/mL. However, the combination of the extract with ampicillin at a concentration of 0.2 mg/mL of extract and 0.01 mg/mL of antibiotic had a bactericidal effect in 3 h, improving the drug’s action time and at much lower concentrations. Some standards of the bioactive compounds reported in coffee and its derivatives were also tested, including caffeine, chlorogenic acid, and caffeic acid, of which caffeine exhibited the greatest bacteriostatic effect individually [66].

### 4.2. Antifungal Activity of Coffee and Its By-Products

In 2022, Badr et al. analyzed the antifungal effect of an isopropanolic extract from spent coffee grounds on fungi that produce mycotoxins, such as those from the genera *Aspergillus*, *Fusarium*, and *Penicillium*. For this, they used the disk diffusion and well diffusion techniques, both of which are fairly standard procedures when investigating compounds with antimicrobial potential. In this case, they found that all the analyzed strains were susceptible to the effects of the bagasse extract, with relatively similar values across the different species treated, as they all exhibited approximately 50% of the effect observed in the antifungal amphotericin B used as a control. Additionally, an experiment was conducted to assess the reduction in mycelial growth of two toxin-producing strains from the *Aspergillus* genus. For this, liquid culture media were used to simulate the effect of fungal contamination in food as it naturally occurs in the environment. The results showed a concentration-dependent decrease in mycelial growth, meaning that at higher concentrations, up to 4 mg/mL, the inhibitory effect was also greater. Furthermore, the extract’s ability to reduce mycotoxin production, particularly aflatoxins and ochratoxins in liquid media, was evaluated. It was found that the anti-toxin effect was also concentration-dependent: the higher the concentration, the lower the toxin production. The results from this research indicate the potential of spent coffee grounds as a food preservative, helping to maintain product safety by keeping them free from mycotoxins. Additionally, it offers the possibility of reusing an agro-industrial byproduct that is usually considered a source of environmental pollution [67].

Barbero López et al. (2020) conducted a study to evaluate the valorization of coffee silverskin as a material with antifungal properties, focusing on its application in wood preservation, as wood is commonly infected by fungi and traditional preservatives are often harmful to the environment. In this study, they used an aqueous extract of coffee silverskin and tested its antimicrobial activity on three fungal strains that cause wood rot: *Rhodonia placenta*, *Gloeophyllum trabeum*, and *Trametes versicolor*. In the antifungal activity tests, the authors found that the 3% silverskin extract was able to inhibit the growth of all three fungi by more than 60%. Toxicity tests were also carried out to assess whether the silverskin extract is truly viable for use as an adjunct to wood preservatives without causing negative environmental effects. The acute ecotoxicity test of the extract was performed on photoluminescent bacteria of the species *Aliivibrio fischeri*, aiming to observe a reduction in bioluminescence after a 30 min period. This analysis found that the extract had relatively low ecotoxic effects, with an EC50 (50% effective concentration) of 2661 mg/L and an EC20 (20% effective concentration) of 1172 mg/L, whereas a common copper-based wood preservative showed higher ecotoxicity, with an EC50 of 19 mg/L and an EC20 of 12 mg/L. In this case, EC50 and EC20 are measures used to evaluate the toxicity of a chemical compound, where lower values indicate higher levels of toxicity. The authors suggest that silverskin extract could function as an additive or a complement to traditional wood preservatives [68].

Calheiros et al. (2023) conducted a study to evaluate the antifungal activity of extracts from coffee bagasse, both caffeinated and decaffeinated, focusing primarily on pathogenic fungi that infect human skin. To determine fungal susceptibility to the extract, they performed the commonly used MIC test (minimum inhibitory concentration) using the broth microdilution technique. This study focused on yeasts and filamentous fungi. They also calculated the minimum fungicidal concentration (MFC), which estimates the lowest concentration of the tested compound that can produce 99% fungal cell death. The strains most susceptible to the analyzed extracts were *Candida krusei*, *Candida parapsilosis*, *Trichophyton mentagrophytes*, and *Trichophyton rubrum*, with the extract from caffeinated coffee bagasse proving to be particularly more effective than that from decaffeinated coffee, which resulted in higher MIC values. Both extracts exhibited fungicidal effects on *T. mentagrophytes* and *T. rubrum*. To attempt to elucidate the mechanism of action of the coffee bagasse extracts on the fungal cell membrane, they evaluated ergosterol and chitin content after applying the extracts and compared it to untreated fungal cells. These compounds are basic structural components of fungal cell membranes, and their reduction or loss indicates the level of damage caused by the extracts. In this test, significant differences (*p* < 0.01) were found in ergosterol and chitin percentages only for *C. parapsilosis* when applying the caffeinated coffee bagasse extract. Notably, the ethanolic coffee bagasse extract produced the best results compared to aqueous extracts [69].

Sangta et al. (2021) sought to find ways to valorize coffee pulp, as this agro-industrial waste is produced in large quantities and its disposal poses a challenge. For this study, the authors used a methanolic extract of coffee pulp. Various concentrations of the extract were selected to evaluate its antifungal activity against agriculturally important fungi, particularly horticultural pathogens. These concentrations were 0.01, 0.03, 0.05, 0.1, and 0.5 g/mL. The antifungal effect observed in this study was concentration-dependent, meaning that higher concentrations resulted in greater inhibition of fungal growth in vitro on Petri dish culture media. The 0.5 g/mL concentration was the most effective against *Alternaria brassicicola*, *Pestalotiopsis* sp., and *Paramyrothecium breviseta*, with the latter showing the greatest susceptibility to the coffee pulp extract, exhibiting a 78% reduction in growth. This study proposes and opens the possibility of reusing agro-industrial waste from coffee production for the control of agricultural diseases caused by phytopathogenic fungi [70].

### 4.3. Antiviral Activity of Coffee and Its By-Products

Coffee, caffeine, and naturally caffeinated beverages are well known for their biological capacity and ability to confer various health benefits, including disease prevention. The alkaloid caffeine present in coffee extracts has demonstrated antiviral activity against herpes simplex virus type 1 [71]. In a study by Batista et al. (2015), it was found that caffeine inhibited hepatitis C virus (HCV) replication in a dose-dependent manner at non-cytotoxic concentrations, with an IC_50_ value of 0.7263 mM after 48 h of incubation [72]. Additionally, caffeine consumption from coffee has been associated with a reduction in viral load in hepatitis C patients and an increased tolerance to peginterferon and ribavirin treatment [73]. In addition to caffeine, the coffee extracts of coffee beans contain significant amounts of chlorogenic acid, which are the major soluble phenolic compounds (Figure S2b). A single cup of coffee may contain 70–350 mg of chlorogenic acid [74]. Consequently, chlorogenic acids and their hydrolysates, which are the main components of the phenolic fraction of coffee beans, have been extensively studied for their antiviral properties [74].

Naturally caffeinated beverages have also been recognized for their broad health benefits. Caffeic acid has been found to have an antiviral effect against the DNA virus (herpes virus HSV) and the non-enveloped RNA virus (polio virus), suppressing virus-infected cell degeneration [75]. Furthermore, regular coffee consumption (more than three cups per day) has been associated with antiviral activity in the human immunodeficiency virus (HIV) and the hepatitis C virus (HCV) [76,77]. According to Shen et al. (2018), caffeic acid can inhibit HCV replications through the induction of heme oxygenase-1, which triggers the antiviral response involving interferon-alpha (IFNα). This antiviral effect is attenuated when HO-1 activity is inhibited by SnPP (an HO-1 inhibitor) [78]. Additionally, Tanida et al. (2015) found that caffeic acid inhibited the initial stage of HCV infection (i.e., between virion entry and translation of the RNA) [79].

In vitro and in vivo studies using crude coffee beans extracts rich in chlorogenic acid, quinic acid, and caffeic acid, have demonstrated substantial inhibitory activity against hepatitis B virus DNA (HBV-DNA) replication in HepG2.2.15 cells [80]. Other studies using aqueous extracts of coffee have shown a notable suppressive effect on hepatitis B virus surface antigens. This biological activity was closely related to the presence of caffeic acid derivatives, especially chlorogenic acid. The suppression of HBV-DNA replication caused by chlorogenic and quinic acids may involve mechanisms such as the inhibition of pgRNA transcription, the capsid encapsulation process, and the disruption of polymerase transcription activity. Additionally, caffeic acid may interfere with the viral maturation pathway [74,81].

The antiviral activity of coffee extract derivates has also been studied in respiratory viruses. Moderate coffee consumption, including decaffeinated coffee, could offer a new guideline for the prevention of SARS-CoV-2 infection. Coffee compounds have been shown to inhibit the binding of the SARS-CoV-2 spike protein to the host ACE2 receptor and reduce the activity of TMPRSS2. Based on the results, researchers suggest that incorporating coffee into the diet may serve as a preventive strategy in the post-COVID-19 era [10].

The Center for Disease Control and Prevention (CDC) recommends caution when consuming caffeine, particularly in energy drinks, due to potential adverse effects at high doses (CDC, 2024). While moderate caffeine intake from coffee may offer health benefits, excessive consumption can lead to overstimulation and cardiovascular issues, potentially outweighing any antiviral effects [82].

Coffee by-products, such as coffee leaves, coffee pulp, coffee husks, silver skin, spent coffee grounds, and other manufacturing industry products, reveal an excellent profile of biological compounds. Behne et al. (2023) identified 5-caffeoylquinic acid (5-CQA) and 3,5-dicaffeoylquinic acid (3,5-DCQA) as the predominant chlorogenic acids in coffee by-products [83]. On the other hand, Wu et al. (2022), found that concentrated coffee leaf extract applied to the skin surface could block SARS-CoV-2 from entering cells more effectively than 75% ethanol, a standard disinfectant recommended by the World Health Organization (WHO) for inactivating SARS-CoV-2 [84].

Most antiviral research has focused on coffee extracts, specifically on their phenolic and alkaloid compounds. Fewer studies have explored the antiviral properties of coffee by-products. Given the growing threat of viral infections, particularly respiratory viruses such as COVID-19, there is an urgent need for alternative strategies to mitigate the transmission of viral variants (i.e., SARS-CoV-2 variants). In this context, exploring the antiviral potential of coffee by-products could provide a viable and sustainable approach to combat viral diseases.

## 5. Regarding the Future of Coffee Residues as a Sustainable Alternative for Antimicrobials Used in Agronomic Practices

As discussed throughout this work, the coffee industry generates a large amount of waste products that contribute to environmental pollution. However, all these residues retain many of the phytochemical properties of the original coffee plant and can therefore be revalued and utilized in various sectors. This review has analyzed articles focused on the reuse of these wastes as a source of bioactive compounds, which exhibit antimicrobial properties. Nevertheless, these studies have been primarily directed toward the healthcare and food sectors, overlooking other areas or sectors that could also benefit from these properties. One such area is the agricultural sector, which is commonly affected by bacterial, fungal, and viral phytopathogens that threaten crop integrity. The usual treatments for these pathogens are often ineffective or harmful to the environment or public health.

Bacterial phytopathogens, in particular, cause losses of up to a billion dollars annually, primarily affecting fruit crops such as pear and apple orchards or even vineyards. Antibiotics are commonly used as treatment or prevention for diseases caused by these microorganisms, with the most used in this sector being streptomycin, gentamicin, oxytetracycline, kasugamycin, and oxolinic acid. Of these, kasugamycin is the only antibiotic not used in the healthcare or veterinary sectors [85]. This presents a significant issue, as bacteria can acquire resistance genes and mechanisms and transfer them to other bacteria that lack them. This is relevant because the use of healthcare antibiotics in agriculture may promote the development of antibiotic resistance in phytopathogenic bacteria, which, when interacting with human or animal pathogens in the environment, could transfer this resistance, thus affecting the efficacy of these medications in clinical and health-related conditions. This underscores the need to seek new substances with bacteriostatic or bactericidal effects, as it is expected that most of the antibiotics currently in use will become obsolete in treating many diseases in the future. Antibiotics are typically applied to crops as ingredients in pesticides, administered preventively or after disease detection. However, there is little control over the actual amount of antibiotics being applied, as it is a poorly regulated process. Despite the belief that their use in agriculture is minimal, this lack of control suggests a wider spread of these drugs than anticipated. Bacterial resistance to streptomycin in phytopathogenic strains has been reported since the 1940s, shortly after its approval for use in crops in 1943. America and Asia are the only continents with authorization for the use of antibiotics in controlling bacterial diseases in crops [6,85,86].

The use of antibiotics in crops is so poorly controlled that many farmers apply them to nematode or mite infestations, which are not affected by these substances designed specifically for bacterial control. As a result, these antibiotics are being unnecessarily exposed to the environment, potentially affecting the persistence and development of soil microflora, which promote plant growth and facilitate nutrient absorption. Another method to mitigate the impact of bacterial diseases is the use of copper salts. However, this also poses a problem, as it promotes the development of bacteria resistant to this metal. It has been observed that metal-resistant bacteria are more likely to acquire antibiotic resistance genes, meaning this cannot be considered an optimal alternative either [6,86].

On the other hand, fungicides are a class of pesticides used in the agricultural sector to prevent or stop diseases caused by fungi in cultivated plants. The most used compounds as fungicides in agriculture are copper, dithiocarbamates, and triazoles/diazoles. Of these, only triazoles/diazoles are used to treat fungal infections in the healthcare and veterinary sectors, in addition to being widely used in Europe to control fungal diseases in crops. Globally, fungicides are the second most widely used class of pesticides in agricultural disease control, accounting for around 23% of pesticide sales [6,87]. Like antibiotics and other antimicrobial compounds, fungicides also promote the evolution of microorganisms and the development of resistance to these compounds, which, in the case of medications also used in human and animal medicine for the same purpose, represents a problem nearly as serious as bacterial resistance [6]. This highlights the importance of seeking alternative compounds to mitigate the impact of fungal infections in crops.

In the case of viral phytopathogens, no effective treatment has been found to mitigate the impact of the diseases they cause in crops. In other words, there is no specific substance with a virucidal effect. Instead, strategies have been employed to help manage the associated plant pathologies. Viruses are intracellular pathogens that typically reach plants with the help of vectors, mostly insects like flies or aphids. These vectors acquire the viruses when they come into contact with an infected plant and spread the pathogen when they “deposit” the virus in a healthy plant [88]. The most commonly used strategy for controlling viral infections in crops is pest control, which involves using products to prevent potential virus-carrying vectors from approaching the plants, such as insecticides. However, this technique proves to be ineffective and continues to result in millions of dollars in losses in the agricultural sector. The most effective technique for controlling viral diseases in crops is the use of seeds or plants with the genetic tools necessary to combat the pathology. This means cultivating plants that are genetically resistant to these viruses, ensuring that the disease does not affect them or spread to neighboring plants [89]. Another commonly used strategy is crop rotation, which involves not planting the same crops consecutively, aiming to prevent vectors that may have approached crop one from containing a virus that could infect crop two [89]. Despite the implementation of these strategies, the lack of a specific and precise treatment for the various viral phytopathogens that plague many of the crops that society relies on for food remains a significant problem. None of these strategies are effective enough to fully control the spread of a viral disease.

In this sense, it becomes clear how agriculture could greatly benefit from the bioactive compounds present in coffee products and by-products, which, in various instances, have already demonstrated inhibitory effects on the growth of these three classes of pathogens. The application of bioactive compounds derived from the coffee plant could offer a more effective treatment for controlling viral diseases, as well as an attractive alternative for controlling bacterial and fungal phytopathogens. While there are specific and well-defined treatments for bacterial and fungal infections, they address one issue but jeopardize the effectiveness and efficiency of medications used in the healthcare and veterinary sectors.

## 6. Conclusions

This review highlights the significant antimicrobial potential of coffee and its by-products, attributed primarily to bioactive compounds such as chlorogenic and caffeic acids. These compounds have been shown to be effective against a range of bacterial, fungal, and viral pathogens, offering promising solutions for combating antimicrobial resistance in healthcare and agriculture. In agriculture, coffee-derived compounds could serve as eco-friendly alternatives for controlling crop diseases, addressing the rising resistance to antibiotics and fungicides. The growing challenge of microbial resistance underlines the urgency of exploring natural sources such as coffee for the development of new antimicrobial agents. The reuse of coffee residues aligns with global efforts to enhance sustainability and reduce waste, supporting a circular economy. The valorization of residues generated during coffee processing, such as pulp, mucilage, husk, and spent grounds, presents a sustainable alternative for mitigating environmental pollution while harnessing their biological activity for practical applications. Future research should focus on optimizing extraction techniques and expanding the applications of these compounds across diverse sectors, particularly in agriculture, where the use of eco-friendly alternatives is increasingly critical. We believe that coffee and its by-products represent an invaluable resource for advancing antimicrobial strategies, fostering sustainability, and promoting environmental stewardship. Their integration into healthcare and agricultural practices holds innovative potential, meriting continued investigation and development.

## Figures and Tables

**Table 1:** Chemical composition, bioactive compounds, and percentage of coffee cherry by by-products.

By-Products	Chemical Composition	Bioactive Compounds	Percentage of the Coffee Cherry	Image
Pulp	Carbohydrates Soluble fibers Proteins Minerals	Chlorogenic acid Caffeine Epicatechin Catechin	40–50%	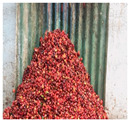
Mucilage	Water Carbohydrates Proteins Pectin’s	Chlorogenic acid Caffeine	14%	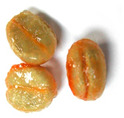
Parchment	(α) cellulose Hemicellulose Lignin Ash	Gallic acid Chlorogenic acid P-cumaric acid Sinapic acid Caffeine	6.1%	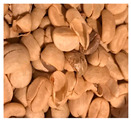
Husk	Carbohydrates Fibers Proteins	Gallic acid Tannic acid Chlorogenic acid Epicatechin Caffeine	50%	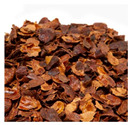
Silver skin	Dietary fiber Polysaccharides Proteins Fats Ash	Caffeine Trigonelline 3-feruloylquinic acid 5-caffeoylquinic acid 3-caffeoylquinic acid Chlorogenic acid P-cumaric acid Melanoidins	4.2%	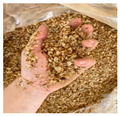
Spent coffee grounds	Polysaccharides Proteins Minerals Fats Dietary fiber Vitamin E Lignin	Chlorogenic acid Caffeic acid Gallic acid Ferulic acid Ellagic acid P-coumaric acid Protocatechuic Tannic acid Catechin Epicatechin Quercetin Rutin Trigonelline Caffeine Melanoidins	90% of the initial coffee beans	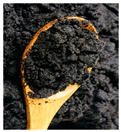

## Data Availability

The original contributions presented in the study are included in the article/Supplementary Materials, further inquiries can be directed to the corresponding authors.

## Supplementary Materials

The following supporting information can be downloaded at: https://www.mdpi.com/article/10.3390/microorganisms13020215/s1, Figure S1: Molecular structure of hydroxybenzoic acids on coffee: (a) gallic acid, (b) syringic acid; Figure S2: Molecular structure of hydroxycinnamic acids on coffee: (a) chlorogenic acid, (b) caffeic acid; Figure S3: Molecular structure of flavonoids on coffee: (a) catechin, (b) que rcetin; Figure S4: Molecular structure of alkaloids on coffee: (a) caffeine, (b) trigonelline; Figure S5: Molecular structure of diterpenes in coffee: (a) kahweol, (b) cafestol.
