# Supplementary figures and images for: The Antimicrobial Effects of Coffee and By-Products and Their Potential Applications in Healthcare and Agricultural Sectors: A State-of-Art Review
Source: Microorganisms. 2025 Jan 21;13(2):215. doi: 10.3390/microorganisms13020215 (PMC11857841; doi:10.3390/microorganisms13020215)

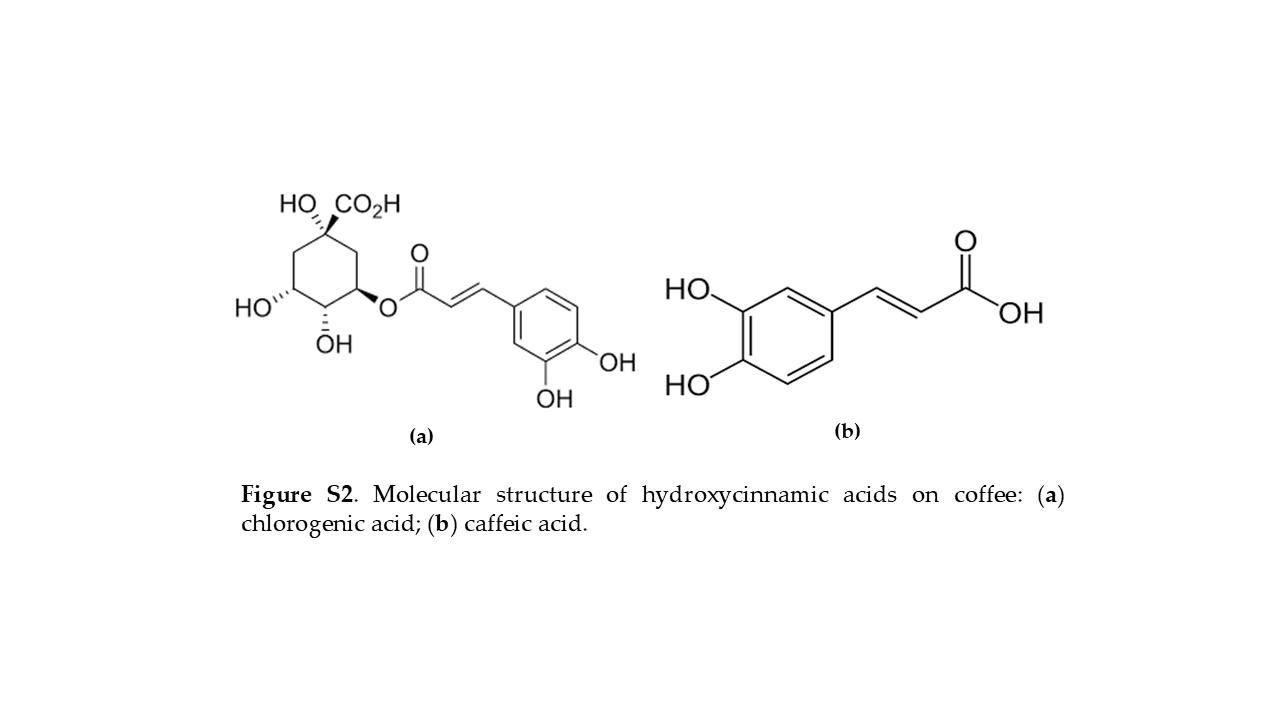

Supplement: Supplementary file 1 [file microorganisms-13-00215-s001.zip › Fig S2-300.tif]

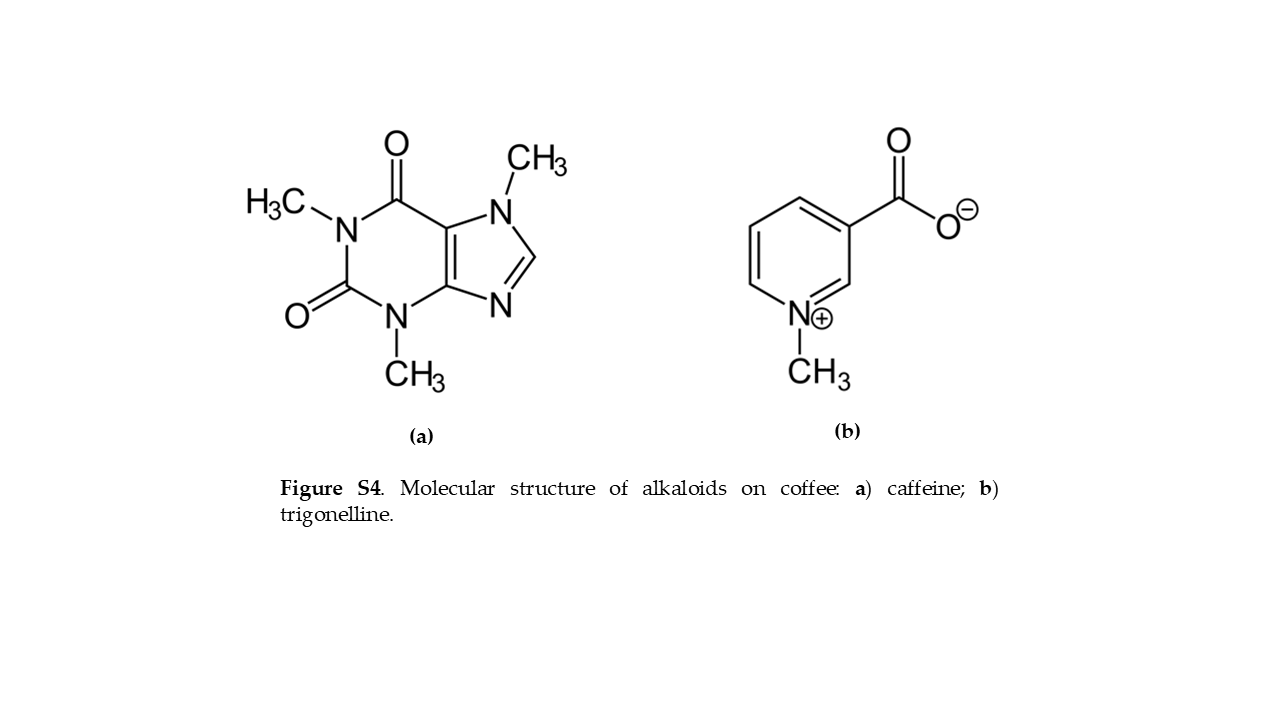

Supplement: Supplementary file 1 [file microorganisms-13-00215-s001.zip › Fig S4-300.tif]

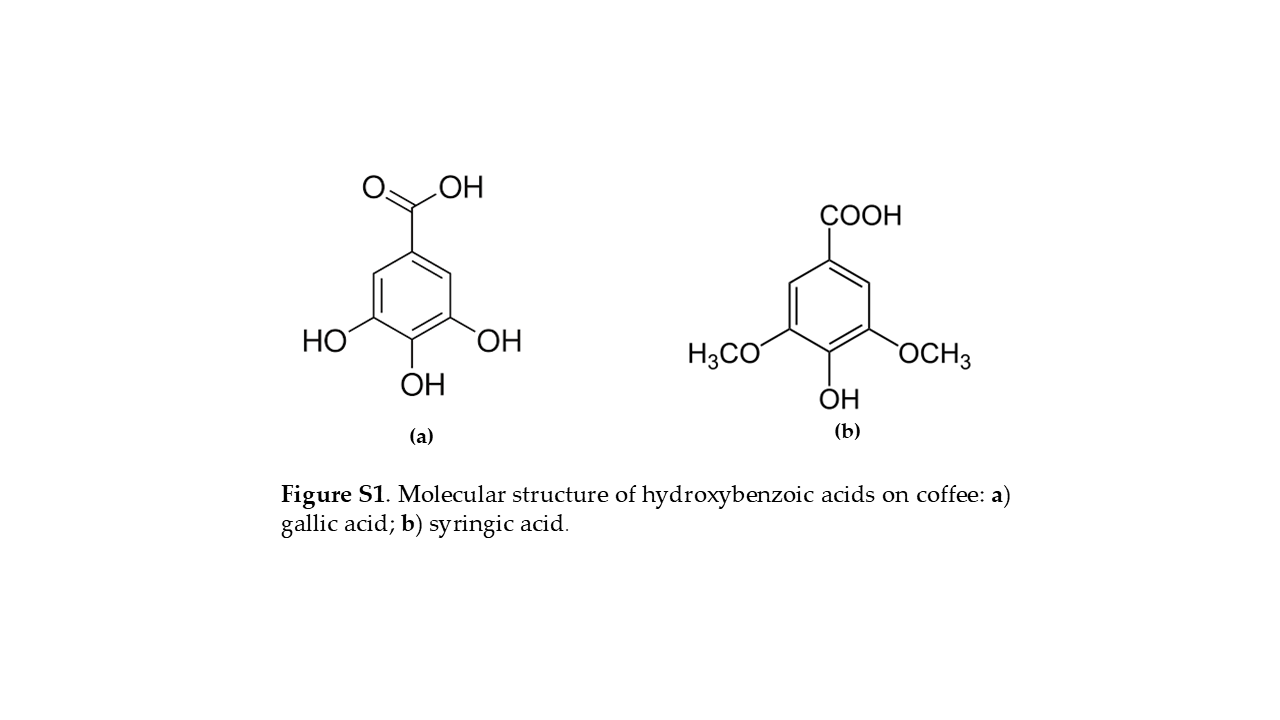

Supplement: Supplementary file 1 [file microorganisms-13-00215-s001.zip › Fig S1-300.tif]

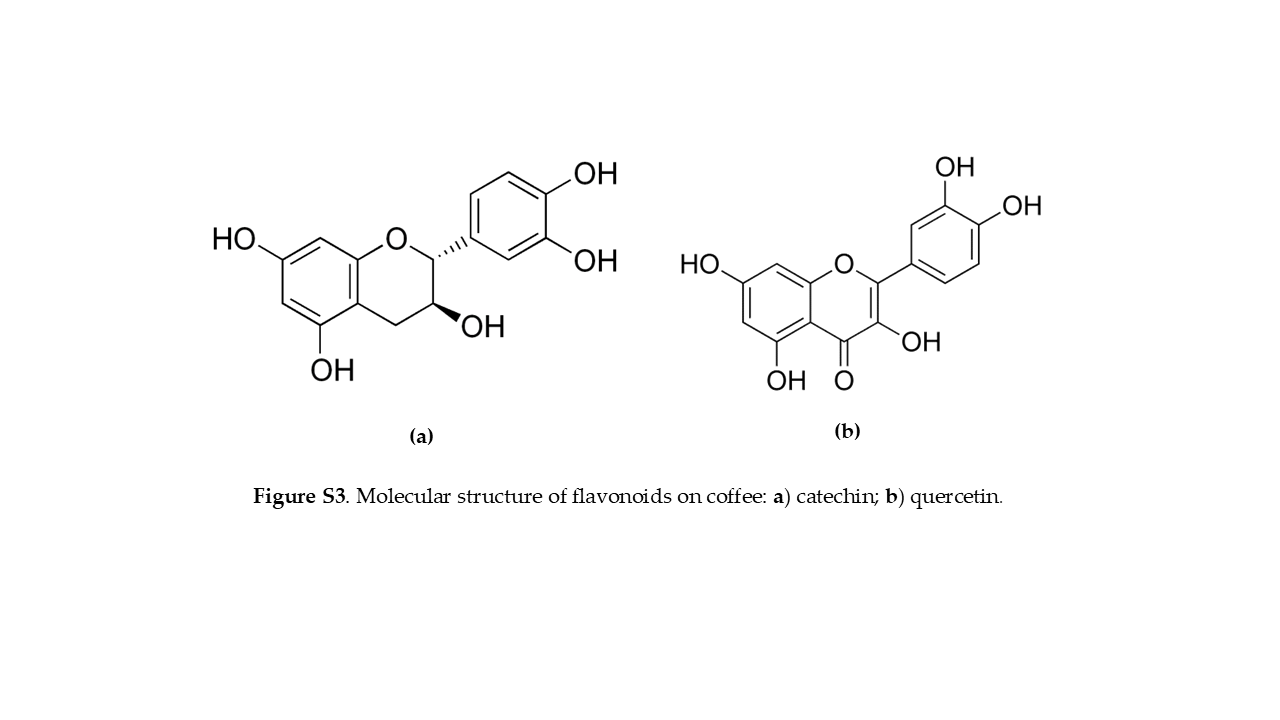

Supplement: Supplementary file 1 [file microorganisms-13-00215-s001.zip › Fig S3-300.tif]

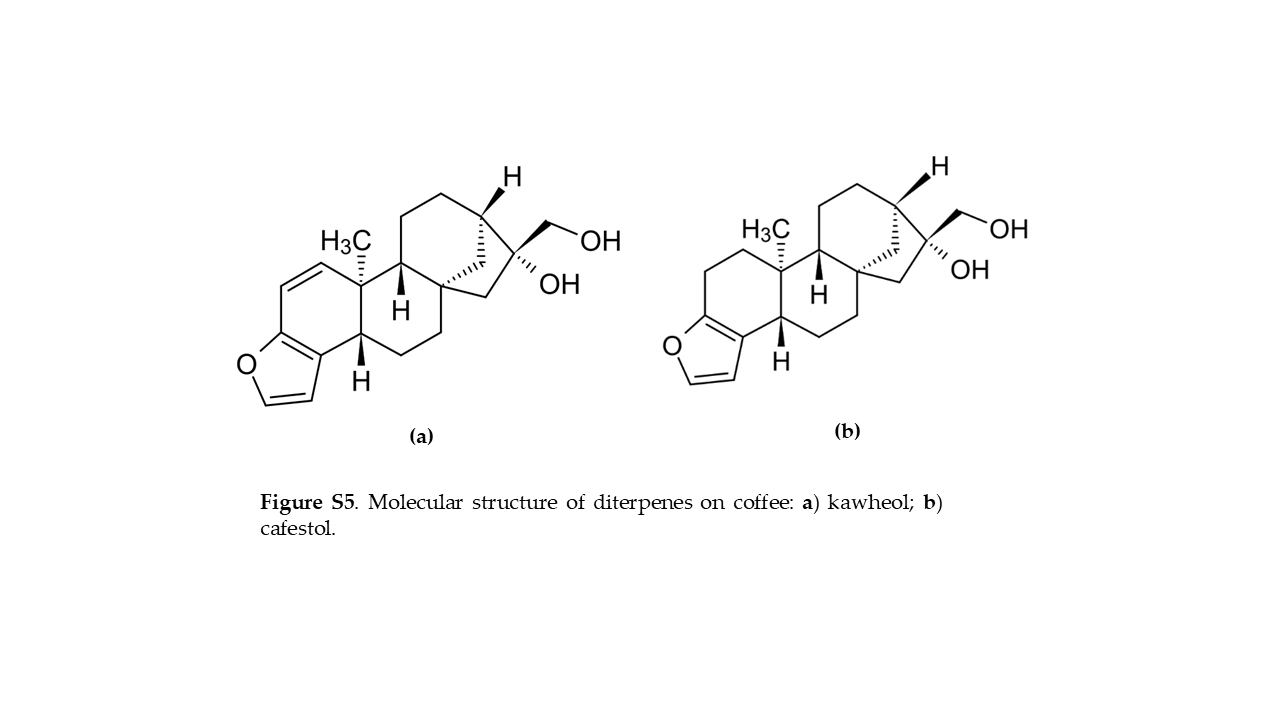

Supplement: Supplementary file 1 [file microorganisms-13-00215-s001.zip › Fig S5-300.tif]
